# *In vivo* and *in vitro* biophysical properties of hair cells from the lateral line and inner ear of developing and adult zebrafish

**DOI:** 10.1113/jphysiol.2013.265108

**Published:** 2014-03-27

**Authors:** Jennifer Olt, Stuart L Johnson, Walter Marcotti

**Affiliations:** Department of Biomedical Science, University of SheffieldSheffield, UK

## Abstract

Hair cells detect and process sound and movement information, and transmit this with remarkable precision and efficiency to afferent neurons via specialized ribbon synapses. The zebrafish is emerging as a powerful model for genetic analysis of hair cell development and function both *in vitro* and *in vivo*. However, the full exploitation of the zebrafish is currently limited by the difficulty in obtaining systematic electrophysiological recordings from hair cells under physiological recording conditions. Thus, the biophysical properties of developing and adult zebrafish hair cells are largely unknown. We investigated potassium and calcium currents, voltage responses and synaptic activity in hair cells from the lateral line and inner ear *in vivo* and using near-physiological *in vitro* recordings. We found that the basolateral current profile of hair cells from the lateral line becomes more segregated with age, and that cells positioned in the centre of the neuromast show more mature characteristics and those towards the edge retain a more immature phenotype. The proportion of mature-like hair cells within a given neuromast increased with zebrafish development. Hair cells from the inner ear showed a developmental change in current profile between the juvenile and adult stages. In lateral line hair cells from juvenile zebrafish, exocytosis also became more efficient and required less calcium for vesicle fusion. In hair cells from mature zebrafish, the biophysical characteristics of ion channels and exocytosis resembled those of hair cells from other lower vertebrates and, to some extent, those in the immature mammalian vestibular and auditory systems. We show that although the zebrafish provides a suitable animal model for studies on hair cell physiology, it is advisable to consider that the age at which the majority of hair cells acquire a mature-type configuration is reached only in the juvenile lateral line and in the inner ear from >2 months after hatching.

## Introduction

Hair cells are specialized mechanosensory receptors in vertebrates that detect and process auditory and vestibular information with remarkable precision, fidelity and efficiency (Schwander *et al*. 2010). Most of the current understanding of how sensory signals are encoded by hair cells has been obtained using mice and other mammals, mainly because of their relatively frequent reproductive cycles and the availability of mouse models for different forms of hearing loss and deafness in humans (Lenz & Avraham, 2011). However, the zebrafish (*Danio rerio*) is being used increasingly to study the genetic basis of hearing and deafness in a process that started with large-scale mutagenesis screens (Nicolson *et al*. 1998; Grunwald & Eisen 2002; Nicolson, 2005). From a functional perspective, the zebrafish also represents an ideal model with which to investigate the physiological mechanisms underlying hair cell function and development *in vivo*, which is extremely difficult to do in mammals. Zebrafish lateral line hair cells are accessible for direct patch clamp experiments and the transparency of the animal during the first 4 weeks post-fertilization (wpf) means that cells can be visualized with the use of genetically targeted fluorescent proteins (Dreosti & Lagnado, 2011). The use of the zebrafish as a model is currently limited by poor understanding of the biophysical properties of its hair cells. Recently, it has been shown that electrophysiological recordings from zebrafish hair cells can be obtained from the lateral line of anaesthetized fish (Ricci *et al*. 2013) and from enzymatically isolated cells from the inner ear (Haden *et al*. 2013). However, these studies provided only preliminary data and nothing is known about how the biophysical properties of hair cells develop and how they relate to the findings of previous studies in mammals.

In zebrafish, hair cells are present in both the lateral line and inner ear. The lateral line includes anterior (head) and posterior (tail) regions that can sense motion frequencies of up to 200 Hz (Ghysen & Dambly-Chaudiere, 2004). The hair cells are clustered in rosette-like structures termed ‘neuromasts’ (Sheets *et al*. 2012). Primary neuromasts are initially deposited by a primordium along the length of the fish during the first 2 days post-fertilization (dpf) and this is followed by three more waves of outgrowth (Pujol-Martí & López-Schier, 2013). Hair cells are supposedly functional a few hours after deposition, but there is evidence for a developmental gradient within a neuromast (Kindt *et al*. 2012). Within the inner ear, the semicircular canals and utricle have a vestibular function, the saccule is required for hearing [up to 4 kHz (Higgs *et al*. 2002)] and the lagena presumably senses both sound and motion (Abbas & Whitfield, 2010). During development, the first few hair cells are distinguishable in the utricular and saccular macula at 2 dpf (Haddon & Lewis, 1996) and in the lagena at 21–25 dpf, and more are added continuously during at least the first year (Bang *et al*. 2001). The growing number of hair cells during the first 2 wpf seems to correlate with increases in auditory responses and sensitivity (Lu & DeSmidt, 2013).

Although hair cells from the inner ear and lateral line seem to begin to respond to stimuli within a few days of fertilization (Kimmel *et al*. 1974; Lu & DeSmidt, 2013), it is not known at which point during development their biophysical characteristics reach functional maturity, nor whether this varies in terms of cell position within a sensory macula or among neuromasts. In the present study, we provide a detailed characterization of the electrical properties of hair cells in the otolithic organs and lateral line from larval to adult zebrafish.

## Methods

### Ethics statement

All zebrafish studies were licensed by the UK Home Office under the Animals (Scientific Procedures) Act 1986 and were approved by the University of Sheffield Ethical Review Committee.

### Tissue preparation

For this study we used zebrafish at different stages of development. Under the husbandry conditions in place at the University of Sheffield and using information from previous studies (Kimmel *et al*. 1995; Parichy *et al*. 2009), we classified the developmental stages of zebrafish as follows: larval: from 3 dpf to ∼2 wpf; juvenile: from ∼2 wpf to the point at which fish become sexually mature (3–6 months), and adult: from 6 months onwards. The average lengths of larvae and juvenile zebrafish in our facility are shown in Fig.1*A*.

**Figure 1:**
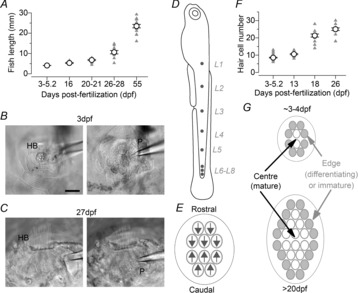
Morphological characteristics of the developing zebrafish lateral line *A*, growth of the zebrafish (*Danio rerio*) during the first 55 days post-fertilization (dpf) under the husbandry conditions used at the University of Sheffield. Numbers of zebrafish measured at each age were 44, 10, 14, 11 and 16, respectively. *B*, *C*, neuromast structure (left; HB, hair bundles) and the hair cell basolateral membrane sealed with a patch pipette (right; P, pipette) in larval (*B*) (3 dpf, L3) and juvenile (*C*) (27 dpf) zebrafish. Scale bar: 10 μm. *D*, lateral view of the larval zebrafish showing the locations of primary neuromasts (L1–L8), each containing hair cells. *E*, diagram of the top view of a neuromast showing hair cells with opposing planar polarities, which define the direction of their mechanoelectrical sensitivity (arrows). The patch pipette was advanced perpendicularly to the neuromast axis of mechanosensitivity allowing easy access to cells of both polarities. *F*, number of hair cells in each neuromast during the first 26 dpf of zebrafish development, which is similar to the stages at which our electrophysiological recordings were obtained in the lateral line. Numbers of neuromasts investigated at each age were 66, 20, 24 and 20, respectively. *G*, diagram of neuromast structure showing the increase in hair cell number according to the degree of differentiation. Note that each neuromast is believed to contain hair cells at different degrees of functional development (Williams & Holder, 2000; López-Schier & Hudspeth, 2006).

In the lateral line, all recordings were performed from the primary neuromasts (L1–L4) originating from the first primordium (primI) (Pujol-Martí & López-Schier, 2013). Electrophysiological recordings from hair cells of larval and juvenile zebrafish were performed mainly in the absence of the commonly used zebrafish anaesthetic tricane methanesulfonate (MS-222; Henry Schein, Inc., Dumfries, UK). In some experiments, MS-222 (0.1%) was used in the solution bathing the fish in order to evaluate possible side-effects of the anaesthetic on hair cell membrane currents. For *in vivo* hair cell recordings in the absence of anaesthetic, larvae (3.0–5.2 dpf) were briefly treated with MS-222 before being paralysed by an injection of 125 μm α-bungarotoxin (α-Btx) (Tocris Bioscience, Bristol, UK) into the heart (Trapani & Nicolson, 2010). Because α-Btx injections could not be performed after 5.2 dpf (zebrafish then become protected animals), older zebrafish were anaesthetized with MS-222, decapitated and immediately washed from anaesthetic with normal extracellular solution. The zebrafish were then transferred to a microscope chamber, immobilized onto a thin layer of sylgard using fine tungsten wire with a diameter of 0.015 nm (larval) and 0.025 nm (juvenile) (Advent Research Materials Ltd, Oxford, UK) and continuously perfused by peristaltic pump with the following extracellular solution: 135 mm (133 mm) NaCl, 1.3 mm (2.8 mm) CaCl_2_, 5.8 mm KCl, 0.9 mm MgCl_2_, 0.7 mm NaH_2_PO_4_, 5.6 mm d-glucose and 10 mm Hepes-NaOH. Sodium pyruvate (2 mm), MEM amino acids solution (50×, without l-glutamine) and MEM vitamins solution (100×) were added from concentrates (Fisher Scientific UK Ltd, Loughborough, UK). The pH was 7.5.

In the inner ear, we investigated hair cells from the three otolithic organs (lagena, sacculus and utricle). Juvenile (7–8 weeks) and adult (>1 year) zebrafish were culled by immersion in a solution containing 0.04% MS-222. Upon cessation of circulation, the fish was transferred into a dissecting chamber containing the normal extracellular solution described above and the inner ear was dissected out. The dissected organ was then transferred into a microscope chamber and immobilized under a nylon mesh attached to a stainless steel ring (Johnson *et al*. 2013). Hair cells in the neuromasts and otolithic organs were viewed with an upright microscope (Olympus OlBX51WI; Olympus KeyMed, Southend-on-Sea, UK) with Nomarski optics.

### Hair cell approach and identification

In the inner ear organs, before hair cells were patched a small portion of their basolateral surface was exposed using a suction pipette (tip diameter: ∼3–4 μm) filled with extracellular solution. This method has been used successfully to record hair cells of the mammalian cochlea (e.g. Marcotti *et al*. 2003a). For the lateral line, the suction pipette was used to remove some skin cells about 20–30 μm from the neuromast in order to avoid any damage to the cupula and the hair bundles. The patch pipette was then advanced towards the cells in the neuromast. In some cases the suction pipette was also used to remove some supporting cells. Hair cells were identified visually by the presence of the hair bundle at the cell's apical surface (larval zebrafish: Fig.1*B*; juvenile zebrafish: Fig.1*C*). Only cells of healthy appearance were used for electrophysiological recordings; thus cells were required to show intact hair bundles, cell membranes with a smooth surface, absence of vacuoles in the cytoplasm and lack of Brownian motion of mitochondria. Hair cells were visualized using a 60× water immersion objective with additional magnification of 1.5× or 2× and 15× eyepieces. Supporting cells were visually distinguished from hair cells based on the absence of hair bundles, the deeper extension of the cell body into the neuromast and, electrophysiologically, by the presence of linear voltage and current responses as previously described (Sugihara & Furukawa, 1996; Ricci *et al*. 2013).

Biophysical responses were recorded from lateral line hair cells positioned in different neuromasts (Fig.1*D*: L1–L4). They were approached perpendicularly to the length of the zebrafish, which allowed access to cells of opposing planar polarity within individual neuromasts (Fig.1*E*). During larval stages, newly formed neuromasts contain about six to 12 hair cells, a number that increases to about 20–30 cells by the juvenile stage (Fig.1*F*). Each neuromast is normally composed of hair cells at different degrees of functional development, and newly differentiating or functionally immature cells are thought to be present towards the edges of the neuromast (Williams & Holder, 2000; López-Schier & Hudspeth, 2006). However, a clear distinction between the central and edge regions was difficult in larval neuromasts due to their very small number of hair cells compared with juvenile neuromasts (Fig.1*G*). Therefore, the distinction between more mature (centre) and immature (edge) cells was made mainly at juvenile stages. The use of the words ‘larval’ (or 3.0–5.2 dpf) and ‘juvenile’ were adopted to adhere to the classical terminology in the literature. However, the age of a given hair cell is unlikely to be linked to the age of the zebrafish (larval or juvenile) because new hair cells are added continually within each neuromast.

### Electrophysiological recordings

Whole-cell patch clamp recordings were primarily performed at room temperature (21–24°C). Calcium current recordings, measurements of exocytosis and some of the voltage responses were conducted at the temperature at which zebrafish are kept (28.5°C). A total of 158 hair cells from the lateral line and 54 hair cells from the inner ear organs were included in this study. Patch clamp recordings from hair cells were generally very stable. In a sample of 135 recordings from both the lateral line and inner ear, the average time was 5.0 ± 0.4 min. This represents an underestimation of the potential recording time because some of the experiments were stopped when all sets of voltage and current clamp protocols had been performed. Moreover, some of the recordings were lost because of involuntary muscle twitching in the fish.

Patch pipettes were made from soda glass capillaries (Harvard Apparatus Ltd, Edenbridge, UK) and had a typical resistance in the extracellular solution of 3–5 MΩ. In order to reduce the fast electrode capacitative transient, the shank of each capillary was coated with surfboard wax (Mr Zog's SexWax; Sexwax, Inc., Carpinteria, CA, USA). For current clamp and K^+^ current recordings, the patch pipette filling solution contained: 131 mm KCl, 3 mm MgCl_2_, 1 mm EGTA-KOH, 5 mm Na_2_ATP, 5 mm Hepes-KOH, and 10 mm sodium phosphocreatine (pH 7.3). For Ca^2+^ current recordings and capacitance measurements, the intracellular solution comprised: 85 mm Cs-glutamate, 20 mm CsCl, 3 mm MgCl_2_, 1 mm EGTA-CsOH, 5 mm Na_2_ATP, 5 mm Hepes-CsOH, 10 mm Na_2_-phosphocreatine, 0.3 mm Na_2_GTP, 15 mm 4-aminopyridine (4-AP), and 20 mm TEA (pH 7.3). Recordings were made with an Optopatch amplifier (Cairn Research Ltd, Faversham, UK). Data acquisition was performed using pClamp software with a Digidata 1322A data acquisition board (Molecular Devices, LLC, Sunnyvale, CA, USA). Recordings were sampled at 5 kHz or 100 kHz, low pass filtered at 2.5 kHz or 10 kHz (8-pole Bessel) and stored on computer for offline analysis (Origin 8.5; OriginLab Corp., Northampton, MA, USA; PClamp 9.2; Molecular Devices, LLC). Membrane potentials in voltage clamp were corrected for the voltage drop across the uncompensated residual series resistance (*R*_s_: 3.5 ± 0.1 MΩ, *n* = 212) and for a liquid junction potential, measured between electrode and bath solutions, of −4 mV for the KCl intracellular solution and −9 mV for Cs-glutamate. Current responses are referred to a holding potential of −84 mV or −79 mV unless specified, and are set to 0-current for comparison between hair cells.

The total number of Ca^2+^ channels per hair cell was estimated using:

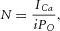
1where *N* is the number of channels, *I_Ca_* is the peak macroscopic Ca^2+^ current, *i* is the single-channel current size, and *P*_o_ is the channel open probability.

### Extracellular superfusion and current isolation

A Ca^2+^-free extracellular solution (including 0.5 mm EGTA) was used to assess the presence of a Ca^2+^-activated K^+^ current (*I*_K,Ca_). The K^+^ channel blocker apamin (300 nM; Tocris Bioscience) was superfused to test whether any Ca^2+^-activated K^+^ current was of the small conductance SK2 type, which is expressed in cochlear hair cells (Marcotti *et al*. 2004). The presence of the h-type current (*I*_h_) was assessed by its resistance to 5 mm BaCl_2_ (Holt & Eatock, 1995). Dihydrostreptomycin (DHS) (0.1 mm or 1 mm; Sigma-Aldrich Co. Ltd, Gillingham, UK) was used to test whether the resting mechanoelectrical transducer current (Marcotti *et al*. 2005) contributes to the hair cell resting membrane potential (Johnson *et al*. 2012). MS-222 (0.1%) was also locally applied to hair cells to investigate its possible effects on membrane currents. Solutions containing drugs were applied through a multi-barrelled pipette positioned close to the preparation. The A-type K^+^ current (*I*_A_) was isolated using a voltage protocol as previously described (Norris *et al*. 1992).

### Statistical analysis

Statistical comparisons were made using the two-tailed Student's *t* test or, for multiple comparisons, one-way ANOVA followed by a Bonferroni *post hoc* test. Values are mean ± s.e.m. A *P*-value of <0.05 indicates statistical significance. In some of the figures statistical significance is indicated by asterisks.

### Phalloidin staining

Adult and juvenile zebrafish were culled using MS-222 (0.04%, until cessation of blood circulation) and decapitated. Heads were placed in a fixative solution containing 4% formaldehyde in 0.1 m sodium phosphate for 2 h at room temperature. Whole otolithic organs were carefully dissected from the labyrinth and washed three times in PBS. The dissected organs were incubated for 2 h in a solution containing 10% heat-inactivated horse serum, 0.1% Triton X-100 (TX-100) and Texas Red-conjugated phalloidin to label F-actin (1 : 300; Molecular Probes, Inc., Eugene, Oregon, USA) in PBS. Following labelling, the tissue was washed three times in PBS and mounted on glass coverslips using Vectashield mounting medium. Nail varnish was used to seal the coverslip onto the slide. Images were taken using an Olympus microscope equipped with a 20× objective and epifluorescence illumination.

### FM1-43 labelling

The entire zebrafish larvae was briefly superfused with a solution containing 6 μm FM1-43 (Gale *et al*. 2001) and hair cells within each neuromast viewed with an upright microscope equipped with epifluorescence optics and FITC filters (excitation 488 nm, emission 520 nm) using the optics described above. Images were captured using a CCD camera (Spot Jr; Toronto Surplus & Scientific, Inc., North York, ON, Canada). Stock solutions of 3 mm FM1-43 were prepared in water. These experiments were performed at room temperature.

## Results

### Biophysical properties of hair cells from the larval zebrafish lateral line

We investigated the electrical properties of hair cells *in vivo* from the lateral line of zebrafish (3.0–5.2 dpf) (Fig.2*A–E*). Hair cells from larval zebrafish had a cell membrane capacitance of 3.6 ± 0.1 pF (*n* = 62). Depolarizing and hyperpolarizing voltage steps from the holding potential of −84 mV elicited voltage-dependent K^+^ currents in all hair cells tested from larval zebrafish neuromasts. Within each neuromast, hair cells exhibited a different combination of K^+^ currents, as evidenced by the variability in the activation and inactivation time course (Fig.2*A*, *B*). A similar combination of K^+^ currents in hair cells was obtained when zebrafish were recorded in the presence (Fig.2*A*) or absence [Fig.2*B* (paralysed with α-Btx)] of the anaesthetic MS-222. We further verified that MS-222 did not affect K^+^ currents in hair cells from larval zebrafish by locally superfusing cells during voltage clamp recordings in paralysed zebrafish (Fig.3). Examples of K^+^ currents recorded from a hair cell (4 dpf zebrafish) before and during the superfusion of 0.1% MS-222 are shown in Fig.3*A* and *B*, respectively. The steady-state values of the K^+^ currents (Fig.3*A*, *B*) were used to generate current–voltage (*I*–*V*) curves. The similar peak and steady-state amplitudes (Fig.3*D*) and steady-state : peak ratio (Fig.3*E*) of the outward K^+^ current indicate that MS-222 does not influence the large variability in the current profile observed in hair cells from larval zebrafish (Fig.2*A*, *B*).

**Figure 2:**
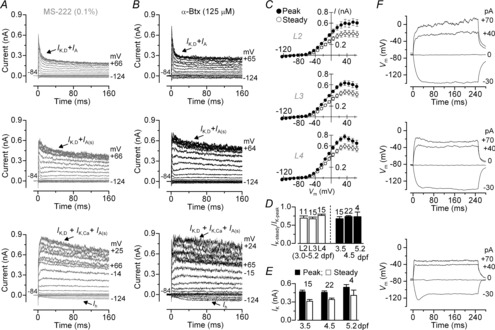
Potassium currents and voltage responses in hair cells from the larval zebrafish lateral line *A*, *B*, examples of K^+^ current recordings from hair cells in different neuromasts (L2–L4) in the presence of the anaesthetic MS-222 (*A*) and when fish were paralysed with α-bungarotoxin (α-Btx) (*B*). Note that the three different current phenotypes were seen in hair cells within each neuromast from larval zebrafish and were unaffected by the presence of MS-222. Currents were elicited by depolarizing and hyperpolarizing voltage steps in 10 mV nominal increments from the holding potential of −84 mV to the various test potentials shown by some of the traces. At this age range (3.5–5.2 dpf) most of the hair cells (about 80%) express *I*_K,D_, *I*_K,Ca_ and a small *I*_A_. *I*_h_ was present only in about 34% of cells and a large *I*_A_ (top panels) was only seen in a small proportion of cells (about 14%). *C*, average peak and steady-state (measured at 160 ms) *I*–*V* curves from hair cells in neuromasts L2–L4. *I*–*V* curves include all recordings (with MS-222 and α-Btx) obtained in each of the three neuromasts investigated, including those in *A* and *B*. *D*, average steady-state : peak amplitude ratio for the outward K^+^ current at 0 mV recorded from hair cells in the three neuromasts (open bars) or as a function of age in all neuromasts (filled bars). *E*, average peak and steady-state amplitudes for the outward K^+^ current at 0 mV recorded from hair cells as a function of age in all neuromasts. *F*, voltage responses to different current injections recorded from hair cells with current responses matching those in *A*. All recordings were performed at room temperature.

**Figure 3:**
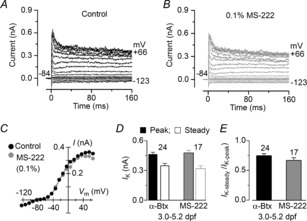
Potassium current recordings from lateral line hair cells of the larval zebrafish before and during local application of MS-222 *A*, *B*, examples of K^+^ currents recorded from a hair cell (4 dpf) before (*A*) and during (*B*) the superfusion of 0.1% MS-222. Currents were elicited as described in Fig.2. *C*, steady-state (measured at 160 ms) *I*–*V* curves obtained from the recordings shown in *A* and *B*. Note that MS-222 did not affect the K^+^ current amplitude, voltage or time dependence. *D*, average peak and steady-state amplitude of the K^+^ current at 0 mV, including those shown in *A* and *B*. Recordings are from hair cells of the larval zebrafish neuromast (3.0–5.2 dpf) in the presence (MS-222) and absence (α-Btx) of the anaesthetic. *E*, average steady-state : peak amplitude ratio for the outward K^+^ current at 0 mV from the hair cells used in *D*.

In hair cells from larval zebrafish, the different combinations of K^+^ currents included several ionic currents that were isolated (data not shown) using either a voltage protocol or a pharmacological approach (see Methods). These currents included a rapidly activating and inactivating A-type current (*I*_A_), a delayed rectifier current (*I*_K,D_), a hyperpolarization-activated K^+^-Na^+^ current (*I*_h_) and a large conductance Ca^2+^-activated K^+^ current (*I*_K,Ca_), which resembled those previously described in the inner ear of the goldfish (Sugihara & Furukawa, 1989, 1996) and frog (Masetto *et al*. 1994; Holt & Eatock, 1995). All 41 hair cells from larval zebrafish expressed *I*_K,D_ and about 80% of them showed the following current profile: *I*_K,D_, *I*_K,Ca_ and a very small *I*_A_ (*I*_A(s)_) (Fig.2*A*, *B*, middle and bottom panels). About 34% of hair cells expressed *I*_h_. Interestingly, a large *I*_A_ (Fig.2*A*, *B*, top panels) was seen in only seven of 41 hair cells investigated [three from 3 dpf (20% of all 3 dpf cells), three from 4 dpf (14% of all 4 dpf cells), one from 5 dpf (25% of all 5 dpf cells)] and was not co-expressed with *I*_K,Ca_.

The peak and steady-state values of the inward and outward currents (Fig.2*A*, *B*) recorded from all hair cells positioned within each of the three neuromasts investigated (L2–L4: Fig.1*D*) were pooled to generate *I*–*V* curves (Fig.2*C*). The three *I*–*V* curves showed similar overall amplitude and voltage dependence, indicating that the current profiles of hair cells within each neuromast showed similar levels of variability, which is also supported by the comparable ratio between steady-state and peak outward K^+^ current (Fig.2*D*). Similar current variability was also seen when hair cells from all three neuromasts of larval zebrafish were grouped as a function of age (Fig.2*D*, *E*). The different current profiles in hair cells are consistent with the observation that each larval neuromast is likely to contain hair cells at different stages of development [based upon hair bundle function (Kindt *et al*. 2012)]. However, in those experiments in which we were able to determine the exact location of hair cells within 3–4 dpf neuromasts (see Methods and Fig.1*G*), we found a similar current profile (*I*_K_, *I*_A(s)_ and *I*_K,Ca_) between cells positioned at the edge (six of six hair cells) and centre (four of six hair cells; two cells were missing *I*_K,Ca_). This indicates that at early larval stages, the different basolateral current profiles of newly formed hair cells are equally present within and across the different neuromasts and as a function of age.

As the composition of K^+^ currents varied among hair cells, we studied how these changes influenced voltage responses in the cells. Hyperpolarizing and depolarizing current steps in 10 pA increments were applied to hair cells positioned in the different neuromasts. Hyperpolarizing current injection caused large voltage responses (Fig.2*F*), which reflects the absence or very small contribution of the inward current (*I*_h_) (Fig.2*A*, *B*). Depolarizing currents elicited voltage responses that reflected the time course of the different components of outward K^+^ currents (Fig.2*A*, *B*). The large variability in the current profile within each neuromast made it difficult to provide a characteristic resting membrane potential (*V*_m_) based upon the position of the hair cell within or between neuromasts. However, the average *V*_m_ for 3.0–5.2 dpf hair cells was −71.4 ± 1.7 mV (*n* = 27). A crucial factor determining the resting *V*_m_ in hair cells is the depolarizing resting inward mechanotransducer current (Johnson *et al*. 2011), which appears to be present in cells of larval zebrafish (Kindt *et al*. 2012; Ricci *et al*. 2013). We tested whether transduction was likely to be functional in hair cells under our *in vivo* recording conditions by using the styryl dye FM1-43 (see Methods), which is a permeant blocker of the hair cell transducer channel (Gale *et al*. 2001). The use of FM1-43 resulted in the selective labelling of hair cells, indicating the presence of a normal resting transducer current (Fig.4*A*). In order to evaluate the functional implication of the depolarizing resting transducer current, we recorded voltage responses in hair cells during the extracellular application of DHS (Fig.4*B*), a known blocker of the mechanotransducer channel (Marcotti *et al*. 2005). In the presence of 0.1–1.0 mm DHS, the membrane potential of hair cells was significantly more hyperpolarized (by ∼7 mV) than in control or washout conditions (*P *< 0.05 and *P *< 0.01, respectively, paired *t* test) (Fig.4*C*), suggesting that the small fraction of transducer channels open at rest directly contributes to cell resting membrane potential in larval zebrafish.

**Figure 4:**
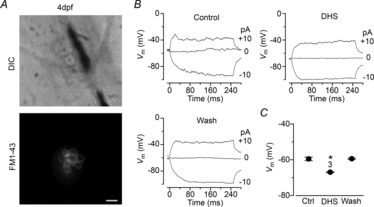
Resting mechanoelectrical transducer current in hair cells from larval zebrafish *A*, differential interference contrast (DIC) and fluorescence images from a 4 dpf larval neuromast (primI) showing that hair cells are labelled with FM1-43, indicating the presence of mechanotransducer channels open at rest (e.g. in the absence of bundle stimulation), which is in agreement with previous findings in mouse hair cells (Gale *et al*. 2001). Scale bar: 10 μm. *B*, voltage responses to current steps shown next to the traces, in control conditions and when the zebrafish (3 dpf, neuromast L4) was exposed to 1 mm dihydrostreptomycin (DHS). Note that blocking the mechanotransducer channels with DHS hyperpolarizes the cell, which was completely reversible after washout of the drug. *C*, average resting membrane potentials measured using the three different conditions described in *B* from three hair cells using 0.1 mm (two cells) or 1 mm DHS (cell in *B*). Recordings in *B* and *C* were performed at 28.5°C.

### Basolateral currents in lateral line hair cells from juvenile zebrafish

We next investigated possible changes in hair cell properties with development by recording their electrical responses in juvenile zebrafish. Hair cells from juvenile zebrafish had a cell membrane capacitance of 3.3 ± 0.1 pF (*n* = 96). Zebrafish are known to become more sensitive to MS-222 with development (Rombough, 2007). Therefore, we tested whether MS-222 affected the biophysical properties of lateral line hair cells from older zebrafish (21–26 dpf). We found that the outward K^+^ current was largely reduced when hair cells were locally superfused with 0.1% MS-222 (Fig.5*A–E*) or when the anaesthetic was constantly present (data not shown). The peak outward K^+^ current appeared to be most sensitive to the anaesthetic (*P *< 0.05) (Fig.5*F*) and both *I*_A_ and *I*_K,Ca_ were largely reduced or blocked.

**Figure 5:**
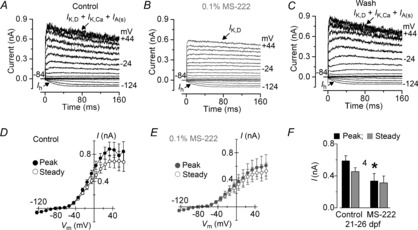
Current recordings from hair cells of the juvenile lateral line before and during local application of MS-222 *A*–*C*, examples of K^+^ currents recorded from hair cells at 21 dpf before (*A*), during (*B*) and after (*C*) local superfusion of 0.1% MS-222. Note that MS-222 mainly blocks the Ca^2+^-activated K^+^ current *I*_K,Ca_ and the small A-type K^+^ current *I*_A(s)_. *I*_h_ was not affected by the anaesthetic. Currents were elicited as described in Fig.2. *D*, *E*, average peak and steady-state *I*–*V* curves for the K^+^ currents recorded before (*D*) and during (*E*) 0.1% MS-222 in four hair cells of 21–26 dpf zebrafish, including the recordings shown in *A* and *B*. *F*, peak and steady-state currents extrapolated at 0 mV before (control) and during (MS-222) the superfusion of the anaesthetic in the same four cells. Note that the peak current was significantly reduced in MS-222. Recordings in *B* and *C* were performed at room temperature.

In order to investigate whether the large variability in the current and voltage responses observed in hair cells from larval (3.0–5.2 dpf) zebrafish reflected their immaturity, we performed similar experiments in juvenile zebrafish (20–37 dpf). By contrast with larvae, the larger neuromasts of juvenile zebrafish (Fig.1*F*, *G*) allowed us to identify the locations of most of the hair cells investigated (37 of 42 hair cells tested). The largest group of hair cells was recorded in 26–29 dpf zebrafish, using 11 cells from the centre and eight from the edge of the neuromast. Figure6*A* and *B* shows typical examples of K^+^ currents and average *I*–*V* curves obtained from hair cells from the centre and edge, respectively. The differences in current profiles were reflected in the characteristic voltage responses (Fig.6*C*, *D*), although average *V*_m_ values were comparable (centre: −69.9 ± 2.5 mV, *n* = 10; edge: −68.3 ± 4.0 mV, *n* = 7; 26–29 dpf). The size of the outward K^+^ current measured at 0 mV was found not to differ significantly between hair cells of larval and juvenile zebrafish (Fig.6*E*), which indicates that the overall number of K^+^ channels is unlikely to increase with development. Note that over the same period, cell membrane capacitance was also similar. However, we did find significant changes in the current profile of cells, with the number of cells expressing *I*_K,Ca_ decreasing and those showing a large *I*_A_ increasing with age (Fig.6*F*). The number of hair cells expressing *I*_h_ remained roughly unchanged. For the two age groups with the biggest hair cell sample size (20–23 dpf and 26–29 dpf), we analysed the K^+^ current expression profile further based on cell position within the neuromast. We found that, similar to hair cells from larval zebrafish, cells from young juvenile zebrafish (20–23 dpf) exhibited a variable current profile across the neuromast, although a larger proportion of cells in the central region expressed a large *I*_A_ (Fig.6*G*, left panel). In 26–29 dpf zebrafish, almost all hair cells in the centre expressed a large *I*_A_ (Fig.6*A*, *G*, right panel), which represents the mature phenotype. By contrast, hair cells from the edge (Fig.6*B*, *G*, right panel) showed a current profile similar to that present in cells from larval neuromasts (Fig.2), which mainly includes *I*_K,D_ and *I*_K,Ca_, and a large *I*_A_ (i.e. ‘mature phenotype’) was only occasionally recorded.

**Figure 6:**
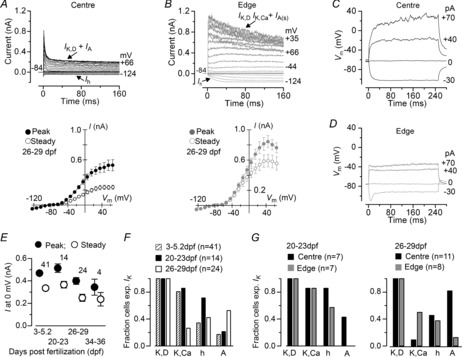
Potassium currents in hair cells from the juvenile zebrafish lateral line *A*, *B*, characteristic K^+^ current recordings (top panels) from lateral line hair cells positioned in the centre (*A*; 29 dpf) and edge (*B*; 28 dpf) of the neuromast (see Fig.1*G*). Bottom panels show the average peak and steady-state *I*–*V* curves obtained from 11 hair cells from the centre (*A*) and eight cells from the edge (*B*). Although all hair cells expressed a delayed rectifier K^+^ current (*I*_K,D_), most of the cells in the centre exhibited a large A-type K^+^ current (*I*_A_) and a small h-type current (*I*_h_). Hair cells from the edge region normally expressed a Ca^2+^-activated K^+^ current (*I*_K,Ca_) and a small *I*_A(s)_. All recordings were obtained from the ventral neuromasts, which originate from the first primordium. Currents were elicited as described in Fig.2*A* and *B*. *C*, *D*, voltage responses to different current injections recorded from the hair cells shown in *A* and *B*. *E*, average peak and steady-state outward K^+^ current, extrapolated at 0 mV from the *I*–*V* curves, measured from hair cells as a function of age. *F*, fraction of hair cells expressing different currents in three stages (age ranges) of zebrafish development. *G*, fraction of hair cells in the centre and at the edge of a juvenile neuromast (left: 20–23 dpf; right: 26–29 dpf) expressing different currents. Recordings were performed at room and 28.5°C.

In addition to hair cells, neuromasts also contain supporting cells, which showed linear current responses (Fig.7*A*, *B*) as previously described in the goldfish sacculus (Sugihara & Furukawa, 1996). Depolarizing and hyperpolarizing current injection caused linear voltage responses (Fig.7*C*), which reflects the absence of voltage-gated ion channels. Supporting cells had a cell membrane capacitance of 4.1 ± 0.3 pF (*n* = 5).

**Figure 7:**
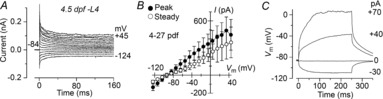
Current and voltage recordings from supporting cells in the neuromast of the larval and juvenile zebrafish lateral line *A*, examples of membrane currents recorded from a supporting cell (4.5 dpf) in neuromast L4 recorded at room temperature. Currents were elicited as described in Fig.2. *B*, average peak and steady-state *I*–*V* curves from five supporting cells (4–27 dpf), including that shown in *A*. *C*, voltage responses recorded in the supporting cell shown in *A*.

### Calcium currents and exocytosis from lateral line hair cells of the larval and juvenile zebrafish

We then investigated the Ca^2+^ current (*I*_Ca_) and induced exocytosis in lateral line hair cells and whether they change as a function of zebrafish development (larval: 3.0–4.5 dpf; juvenile: 17–34 dpf). *I*_Ca_ was isolated from the total membrane current by blocking the K^+^ currents with 4-AP and TEA in the caesium-based intracellular solution (see Methods). Figure8*A* shows a typical example of *I*_Ca_ recorded in hair cells from larval (3.0–4.5 dpf) and juvenile (29–34 dpf) zebrafish in the presence of 2.8 mm extracellular Ca^2+^ at the physiological temperature for the zebrafish (28.5°C). The average *I*–*V* curve measured at the peak *I*_Ca_ (Fig.8*B*) was fitted using the following equation:

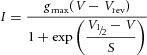
2where *I* is the current, *g*_max_ is the maximum chord conductance, *V* is the membrane potential, *V*_rev_ is the reversal potential of the current, *V*_½_ is the potential at which the conductance is half activated, and *S* is the slope factor that defines the voltage sensitivity of current activation. *I*_Ca_ activated at around −70 mV (defined as 5% of *g*_max_) (Fig.8*B*). The maximum size of *I*_Ca_ was similar between hair cells of different ages (3.0–4.5 dpf: −12.3 ± 1.1 pA, *n* = 6; 17–22 dpf: −13.0 ± 2.9 pA, *n* = 5; 29–34 dpf: −10.5 ± 2.7 pA, *n* = 6; measured near −35 mV). Exocytosis was estimated by measuring changes in cell membrane capacitance (Δ*C*_m_) following depolarizing voltage steps, which is generally interpreted as a sign of neurotransmitter release from presynaptic cells (Moser & Beutner, 2000; Johnson *et al*. 2008, 2013). Figure8*C* shows Δ*C*_m_ recorded in response to a 1 s depolarizing voltage step from 3.0–4.5 dpf and 29–34 dpf hair cells. Despite the similar *I*_Ca_, hair cells of juvenile zebrafish showed a significantly (*P *< 0.001) larger Δ*C*_m_ (6.6 ± 0.6 fF, *n* = 5) compared with those of larval fish (2.3 ± 0.5 fF, *n *= 6). This increase in Δ*C*_m_ with age was already present in hair cells from 17–22 dpf zebrafish (7.7 ± 2.3 fF, *n *= 4, significant at *P *< 0.05 compared with hair cells from larval zebrafish).

**Figure 8:**
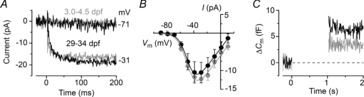
Ca^2+^ currents and neurotransmitter release in lateral line hair cells *A*, Ca^2+^ currents (*I*_Ca_) recorded from hair cells of the larval (3.0–4.5 dpf: grey) and juvenile (29–34 dpf: black) zebrafish lateral line. Currents were elicited by depolarizing voltage steps of 10 mV increments (200 ms in duration) from the holding potential of −79 mV. For clarity only two of the traces are shown. *B*, average peak Ca^2+^ current *I*–*V* curves from 3.0–4.5 dpf (grey) and 29–34 dpf (black) hair cells, including those shown in *A*. The continuous lines are fits using eqn 2. Fitting parameters are (for 3.0–4.5 dpf) *g*_max _= 0.2 nS, *V*_rev_ = 17 mV, *V*_½_ = −48.2 mV and *S* = 6.6 mV, and (for 29–34 dpf) *g*_max _= 0.2 nS, *V*_rev_ = 24 mV, *V*_½_ = −48.3 mV and *S* = 7.1 mV. *C*, changes in membrane capacitance (Δ*C*_m_) recorded from hair cells of larval (3.0–4.5 dpf: grey) and juvenile (29–34 dpf: black) zebrafish. Recordings were obtained in response to 1 s voltage steps from the holding potential of −79 mV to near the peak of *I*_Ca_ (near −30 mV). Recordings were obtained at 28.5°C and using 2.8 mm extracellular Ca^2+^.

### Current and voltage responses elicited from hair cells of the zebrafish lagena

We investigated whether the biophysical properties of hair cells present in the auditory-like (sacculus and lagena) or vestibular-like (utricule and lagena) organs in the zebrafish inner ear (Abbas & Whitfield, 2010) were comparable with those observed in the lateral line. Hair cells in the lagena appear from around 4 wpf (Bang *et al*. 2001), just before *in vitro* recordings could be made at 7 wpf.

The morphology of hair cells in the zebrafish inner ear sensory epithelia has been shown to be heterogeneous (Bang *et al*. 2001). Therefore, we investigated the development of the basolateral membrane properties of hair cells from the juvenile (Fig.9) and adult (Fig.10) lagena positioned in different regions of the posterior portion of the sensory epithelium [Fig.10*A* (centre: red; edge: yellow)]. Hair cells had a cell membrane capacitance of 3.1 ± 0.1 pF (*n* = 36). In the juvenile zebrafish (7 wpf), depolarizing and hyperpolarizing voltage steps revealed variable K^+^ current profiles in hair cells from both the centre and edge of the lagena (Fig.9). Hair cells from the adult (>1 year) lagena, unlike those from the juvenile zebrafish (Fig.9), exhibited only two current profiles (Fig.10*B*, *C*). Depolarizing voltage steps caused slowly developing (centre) and rapidly activating and inactivating (edge) voltage-dependent outward K^+^ currents. Hyperpolarizing steps elicited inward currents that also differed depending on the cell's position within the sensory epithelium. These two types of hair cell [Fig.10*B* (centre), *C* (edge)] resembled those isolated from the goldfish sacculus (Sugihara & Furukawa, 1989, 1996): one cell type expressed *I*_K,D_ and *I*_K1_; the other type mainly showed *I*_A_ and *I*_h_, and possibly a small *I*_K,D_. The current profile seen in hair cells from the centre closely resembled that present in immature mouse cochlear inner hair cells (IHCs) (Marcotti *et al*. 1999, 2003a). The *I*–*V* curves were generated by measuring peak currents (Fig.10*D*). The overall amplitude of the outward K^+^ current in hair cells from the juvenile and adult zebrafish lagena did not change significantly (Fig.10*E*). However, the change in the current profile during development suggests that at 7 weeks the hair cells of the lagena have yet to acquire their mature basolateral membrane current profile.

**Figure 9:**
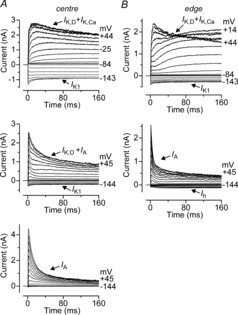
Membrane currents from hair cells of the juvenile zebrafish lagena *A*, *B*, typical K^+^ currents from hair cells positioned in the centre (*A*) and edge (*B*) of the lagena in a 7-week-old zebrafish. Note that the current profile recorded within each region was variable. Currents were elicited as described in Fig.2. In the centre (*A*), four of 11 hair cells exhibited the current profile shown in the top panel, which is typical of adult cells from the central region (Fig.10*B*); seven expressed *I*_A_ (bottom two panels), resembling that present in cells from the edge region of adult cells (Fig.10*C*). In the edge (*B*), two hair cells exhibited the top profile and six expressed *I*_A_ (lower panel), which is characteristic of this region in the adult (Fig.10*C*). Recordings were performed at room temperature.

**Figure 10:**
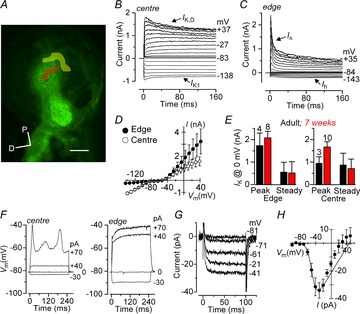
Membrane currents and voltage responses from hair cells of the juvenile and adult zebrafish lagena *A*, phalloidin-stained hair bundles in an adult zebrafish lagena showing the central (red) and edge (yellow) regions of the posterior sensory organ used for the recordings. P, posterior; D, dorsal. Scale bar: 120 μm. *B*, *C*, typical K^+^ currents from hair cells positioned in the centre (*B*) and edge (*C*) of the posterior lagena. Note the different channel profiles. Currents were elicited as described in Fig.2. *D*, average peak *I*–*V* curves from hair cells in the centre and edge, including those in *B* and *C*. Recordings shown in *B*–*D* are from the adult zebrafish. *E*, average peak and steady-state amplitudes of the total outward K^+^ current in the two different regions from adult (>1 year) and juvenile (7 week) (see also Fig.9) zebrafish. *F*, voltage responses showing that hair cells from the centre (left panel), but not the edge (right panel) of the adult lagena show action potentials. *G*, example of Ca^2+^ currents (*I*_Ca_) recorded from a hair cell at the edge of the adult lagena. Currents were elicited as described in Fig.8. *H*, average peak *I*–*V* curve for the Ca^2+^ currents obtained from six hair cells of the adult lagena (from both regions), including that shown in *G*. The continuous line is a fit between −101 mV and −20 mV using eqn 2. Fitting parameters are *g*_max _= 1.6 nS, *V*_rev_ = 43 mV, *V*_½_ = −49 mV and *S* = 6.3 mV. Note that for values more depolarized than −20 mV, the fit was extrapolated. Recordings in *B*–*F* were performed at room temperature and G and H at 28.5°C.

The functional significance of the different K^+^ currents on the physiology of hair cells from the centre and edge was evaluated in current clamp experiments (Fig.10*F*). Hyperpolarizing current steps in 10 pA increments caused small voltage changes in hair cells from the centre, attributable to the large *I*_K1_ (Marcotti *et al*. 1999), and larger voltage responses in cells from the edge. Depolarizing current injections elicited broad action potentials only in hair cells from the centre, which had a current profile similar to spiking IHCs (Marcotti *et al*. 2003a, 2003*b*). A typical example of *I*_Ca_ recorded from hair cells of the lagena in the presence of 2.8 mm extracellular Ca^2+^ and at 28.5°C is shown in Fig.10*G*. The average *I*–*V* curve measured at the peak *I*_Ca_ (Fig.10*H*) was fitted using eqn 2. *I*_Ca_ activated at around −67 mV (5% of *g*_max_) and reached its maximum size at −31 mV (−34 ± 6 pA, *n* = 6). The *V*_m_ of hair cells positioned in the centre (−74.4 ± 2.0 mV, *n* = 8) was similar to that of cells at the edge (−72.5 ± 3.4 mV, *n* = 6).

### Current and voltage responses recorded from hair cells of the zebrafish sacculus and utricle

We then investigated whether the basolateral membrane properties of hair cells from the adult zebrafish (>1 year) sacculus and utricle were comparable with those observed in the lagena. Hair cells positioned in the posterior portion of the saccular sensory macula (Fig.11*A*) expressed K^+^ currents (Fig.11*B–D*) similar to those present in cells from the lagena (Fig.10*B–D*). Saccular hair cells had a cell membrane capacitance of 3.2 ± 0.4 pF (*n* = 8) and a resting membrane potential of −71 ± 3 mV (*n* = 7). The *I*–*V* curves were generated by measuring peak currents (Fig.11*D*). Typical voltage responses of hair cells from the centre and edge of the sacculus (Fig.11*E*) were comparable with those recorded in the lagena (Fig.10*F*), which is consistent with cells from the two sensory organs expressing similar K^+^ currents. Utricular hair cells (Fig.12*A*) showed one main current profile (Fig.12*B*, *C*), which was similar to that present in cells positioned at the edge of the lagena and saccular macula (Figs10*C* and 11*C*, respectively). Consequently, utricular hair cells also showed comparable voltage responses (Fig.12*D*). Hair cells from the utricle had a cell membrane capacitance of 3.2 ± 0.2 pF (*n* = 11) and a resting membrane potential of −71 ± 2 mV (*n* = 8).

**Figure 11:**
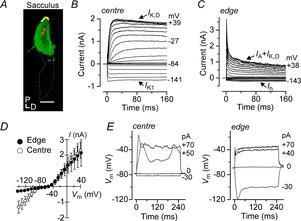
Membrane currents from adult hair cells of the zebrafish sacculus *A*, phalloidin-stained hair bundles of saccular hair cells from the adult zebrafish showing the central (red) and edge (yellow) regions of the posterior sensory organ used for the recordings. The dotted line delineates the entire sacculus, including the unlabelled portion that could not be successfully removed from the inner ear. P, posterior; D, dorsal. Scale bar: 120 μm. *B*, *C*, typical K^+^ currents from hair cells positioned in the centre (*B*) and edge (*C*) of the sacculus. Note the different current profiles, which are similar to those found in the mature lagena (Fig.10). Currents were elicited as described in Fig.2. Note that in the central region, four hair cells showed the profile depicted in *B* and one showed that in *C*. In the edge, all three cells were as in C. *D*, average peak *I*–*V* curves from hair cells in the centre and edge, including those in *B* and *C*. *E*, voltage responses recorded from hair cells positioned in the centre (left) and edge (right) of the sacculus. Recordings were performed at room temperature.

**Figure 12:**
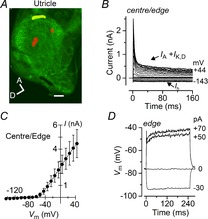
Membrane currents and voltage responses from adult hair cells of the zebrafish utricle *A*, phalloidin-stained hair bundles of the utricular hair cells from adult zebrafish showing the central (red) and edge (yellow) regions used for the recording. A, anterior; D, dorsal. Scale bar: 120 μm. *B*, example of K^+^ currents recorded from a utricular hair cell; nine of 10 hair cells recorded in the centre and edge regions showed the profile in *B* (one cell showed an inward *I*_K1_ instead of *I*_h_). Currents were elicited as described in Fig.2. *C*, average peak *I*–*V* curves from all hair cells in the centre and edge, including that in *B*. *D*, voltage responses recorded from a hair cell positioned at the edge of the utricle. Recordings were performed at room temperature.

## Discussion

In this study, using *in vivo* and *in vitro* recordings, we determined the biophysical properties of hair cells in the lateral line and inner ear of the developing zebrafish. We found that the majority of zebrafish hair cells acquire mature basolateral membrane currents and synaptic neurotransmission only from juvenile stages in the lateral and from >2 months in the inner ear. Based on their complement of ion channels, mature zebrafish hair cells resemble those of other lower vertebrates and, to some extent, hair cells from the immature mammalian vestibular and auditory systems. We demonstrate that zebrafish represent a suitable model for the study of hair cell function, and that the proportion of hair cells showing mature-like basolateral membrane properties increases with zebrafish development.

### Physiological maturation of hair cells in the posterior zebrafish lateral line

The lateral line of the zebrafish is responsible for signalling perceptions of hydrodynamics around the body, which are used for orientation and the coordination of motor behaviour in terms of location, direction and velocity (Bleckmann & Zelick, 2009; Pujol-Martí & López-Schier, 2013). Similarly to the auditory and vestibular systems of lower vertebrates and mammals (Fettiplace & Hackney, 2006), lateral line neuromast hair cells mechanoelectrically transduce motion of their stereociliary hair bundles (Nicolson, 2005). The reduced complexity and the accessibility of the lateral line in the larval zebrafish has driven its use to understand hair cell sensory transduction. However, an important issue concerns whether the biophysical properties of hair cells in the lateral line of the larval zebrafish are mature and comparable with those of auditory and vestibular hair cells in mammals. We found the basolateral K^+^ current profile of hair cells within each neuromast to be quite variable (Figs2 and 6), indicating the presence of cells with differing degrees of functional maturity. Similar degrees of variability were seen in hair cells of different neuromasts at 3.0–5.2 dpf (Fig.2: L2–L4), despite the fact that the most anterior primary neuromast (L1) are deposited 20 h before the last primary neuromasts (L6–L8), and the fish undergoes dramatic changes during these early stages (Kimmel *et al*. 1995; Pujol-Martí & López-Schier, 2013). With development, hair cells show a progressive segregation within a neuromast such that cells positioned in the centre have mature characteristics and those towards the edge retain a more immature phenotype. Although the delayed rectifier K^+^ current (*I*_K,D_) was present in all hair cells investigated, cells from the centre of the juvenile neuromast were mainly characterized by a large A-type K^+^ current (*I*_A_). Hair cells at the edge mainly expressed a large conductance Ca^2+^-activated K^+^ current (*I*_K,Ca_) and a small *I*_A_ (*I*_A(s)_). Only about 34% of cells in both regions expressed the h-type current (*I*_h_). This finding supports previous morphological observations that newly differentiating or functionally immature hair cells mainly originate from the edge of the neuromast (Williams & Holder, 2000; López-Schier & Hudspeth, 2006; Kindt *et al*. 2012). Therefore, the neuromasts of the posterior lateral line require around 2 weeks to reach full maturity, the central region contains mainly mature-like hair cells and the edge consists of newly differentiating cells. This delay in maturation is likely to be restricted to the basolateral membrane properties because hair cells from larval zebrafish show mechanoelectrical transduction at the hair bundles (Trapani & Nicolson, 2010; Kindt *et al*. 2012). This is not surprising given that mature mechanoelectrical transducer currents in the utricle (Géléoc & Holt, 2003) and outer hair cells (OHCs) (Waguespack *et al*. 2007; Lelli *et al*. 2009) have been observed prior to the functional maturation of hair cells in the mammalian vestibular and auditory sensory organs [utricle (Rüsch *et al*. 1998) and OHCs (Marcotti & Kros, 1999)].

### Calcium current and exocytosis at lateral line hair cell ribbon synapses

By contrast with K^+^ currents, the amplitude of the Ca^2+^ current did not change between lateral line hair cells recorded from larval (3.0–4.5 dpf) and juvenile (17–22 dpf and 29–34 dpf) zebrafish. However, hair cells from juvenile zebrafish exhibited more efficient exocytosis at ribbon synapses, requiring less Ca^2+^ for vesicle fusion than cells from larval zebrafish. This finding further supports our observation that most hair cells in larval zebrafish are immature in terms of their basolateral membrane properties.

All lateral line hair cells showed a very small *I*_Ca_ (Fig.8) consistent with that reported in frog and rat vestibular hair cells (Prigioni *et al*. 1992; Bao *et al*. 2003) and with recent data for hair cells of the larval zebrafish lateral line (Ricci *et al*. 2013). The Ca^2+^ current in lateral line hair cells is carried by Ca_v_1.3 Ca^2+^ channels clustered at the presynaptic active zones (Sidi *et al*. 2004; Sheets *et al*. 2012), as has been shown for hair cells in the mammalian auditory and vestibular systems (Platzer *et al*. 2000; Bao *et al*. 2003; Brandt *et al*. 2003). In view of the single Ca_v_1.3 Ca^2+^ channel properties of mature cochlear hair cells [*i* = −0.34 pA; *P_o_* = 0.21 pA (Zampini *et al*. 2013)], the total number of Ca^2+^ channels in lateral line hair cells from juvenile (mature) zebrafish (peak current of −11 pA) is likely to be in the order of about 150 [see eqn 1]. Assuming that all 150 Ca^2+^channels are associated with ribbons (Sidi *et al*. 2004; Sheets *et al*. 2012), each of the four active zones (ribbons with co-localized Ca^2+^ channels) present in these cells (Sidi *et al*. 2004) is likely to contain 38 Ca^2+^ channels. This value is similar to that of previous estimates in the adult bullfrog (Graydon *et al*. 2011), but is about five times smaller than that measured in cochlear hair cells [∼180 Ca^2+^ channels (Zampini *et al*. 2010, 2013)].

Although small, the Ca^2+^ current in lateral line hair cells was sufficient to trigger the fusion of synaptic vesicles (Fig.8) containing glutamate (Obholzer *et al*. 2008). The amplitude of *I*_Ca_ was similar in hair cells from larval and juvenile zebrafish (Fig.8), but in the juvenile was able to release about three times more synaptic vesicles [larva: 62 vesicles; juvenile: 178 vesicles, using a conversion factor of 37 aF/vesicle (Lenzi *et al*. 1999)], indicating an increase in the Ca^2+^ efficiency of neurotransmitter release with development. In the juvenile zebrafish (mature hair cells), 178 vesicles equate to ∼45 vesicles for each of the four active zones (Sidi *et al*. 2004). A similar depolarizing voltage step in mature mouse IHCs has been shown to recruit about 4000 vesicles [∼150 fF (Johnson *et al*. 2005)] and ∼270 vesicles per active zone, which is about six times as many as that in hair cells from the juvenile zebrafish lateral line. The similarity in the proportions of vesicles per Ca^2+^ channel at each ribbon between lateral line (1.2) hair cells and IHCs (1.5) indicates that the efficiency of neurotransmitter release in hair cells is likely to be very similar between the mammalian cochlea and zebrafish lateral line. How the different basolateral membrane properties of developing hair cells affect synaptic signal encoding at the afferent fibre is currently unknown, mainly because we still know little about lateral line function and organization in the adult zebrafish (Nicolson & Trapani, 2011; Haehnel *et al*. 2012; Liao & Haehnel, 2012). However, we do know that the neuromasts and afferent fibres of the zebrafish lateral line undergo extensive growth and reorganization during larval stages that result in a more complex organization in the adult, which is likely to be essential to the fine-tuning of sensitivity to movement direction (Haehnel *et al*. 2012; Liao & Haehnel, 2012).

### Biophysical properties of the zebrafish inner ear hair cells

Our results have demonstrated that the mature zebrafish inner ear sensory organs express two classes of hair cells with distinct current profiles that develop during juvenile stages. Both of these current profiles are characteristic of mature inner ear hair cells. In the lagena and sacculus, which have a primary function in hearing in fish (Abbas & Whitfield, 2010), one population of hair cells mainly expresses the A-type K^+^ and the h-type K^+^-Na^+^ currents that are characteristic of vestibular organs in bird (Masetto & Correia, 1997; Masetto *et al*. 2000) and mouse (Rüsch *et al*. 1998), but absent in hair cells of the mammalian cochlea (Marcotti & Kros, 1999; Marcotti *et al*. 2003a). The second population of cells shows K^+^ currents (*I*_K,D_ and *I*_K1_) that resemble those in the auditory organs of other lower vertebrates, including in the goldfish sacculus (Sugihara & Furukawa, 1989, 1996), and mammals, such as in immature mouse IHCs (Marcotti & Kros, 1999; Marcotti *et al*. 2003). Utricular hair cells, which are responsible for balance in zebrafish, predominantly show one current profile (A-type and h-type currents), which is similar to that of the lagena and sacculus. Although our description of two main current profiles agrees with that of an earlier preliminary study (Knirsch & Rüsch, 2003), a recent study reported a large variation in the current profile of enzymatically isolated hair cells from the zebrafish inner ear and up to six different combinations of K^+^ currents (Haden *et al*. 2013). One possible explanation for this discrepancy may refer to alterations in channel properties when cells are treated with enzyme, which is also suggested by the fact that the A-type current was barely visible and the h-type current was absent in the earlier recordings (Haden *et al*. 2013).

This study provides crucial information on how the sensory hair cells of the zebrafish develop and function and highlights the fact that fish represent an ideal model for investigations into the function of hair cells *in vivo*, which are extremely difficult in mammals. In particular, the zebrafish will represent a useful tool for investigations of synaptic mechanisms at hair cell ribbon synapses because they allow for the ability to combine genetic manipulation with electrophysiology and imaging *in vivo*. However, it is important to consider the following factors: (i) it is advisable that mature-like function in lateral line hair cells is investigated starting from juvenile stages because the proportion of mature cells increases with zebrafish development; (ii) mature-like hair cells in juvenile zebrafish are confined to the centre of the neuromast, whereas no segregation is observed in larval neuromasts; (iii) hair cells with ‘immature’ biophysical characteristics are present at every stage of zebrafish development, although their proportion decreases with age and, in juvenile zebrafish, they are confined to the edge of the neuromast, and (iv) the basolateral characteristics of mature zebrafish hair cells resemble, to some extent, only those in the immature mammalian vestibular and auditory systems.

## Key points

- Sound and balance information is detected and processed by sensory hair cells in the auditory and vestibular organs, respectively.
- The zebrafish represents a potentially powerful model organism in which to investigate sensory encoding by hair cells because of its accessibility for *in vivo* studies and its pliable genetics.
- Our current understanding of hair cell biophysics in the developing zebrafish is very limited.
- In this study, we used *in vivo* and near-physiological *in vitro* recordings to measure basolateral membrane currents, voltage changes and synaptic activity in hair cells in the developing and mature zebrafish.
- We found that the biophysical profile of lateral line hair cells in the zebrafish changes from the larval to the juvenile stage, and that juvenile neuromasts contain a much higher proportion of mature cells.
- These results demonstrate the potential of the zebrafish for investigating the mechanisms of signal encoding and transmission by hair cells.

## Abbreviations

dpf: days post-fertilization
IHC: inner hair cell
OHC: outer hair cell
primI: first primordium
wpf: weeks post-fertilization
